# A case of ulnar deviation (drift) in a patient with chronic rheumatoid arthritis

**DOI:** 10.11604/pamj.2024.48.35.40672

**Published:** 2024-05-30

**Authors:** Amol Madhav Deshpande, Mayuri Amol Deshpande

**Affiliations:** 1 Department of Rachana Sharir, Mahatma Gandhi Ayurved College Hospital and Research Centre, Datta Meghe Institute of Higher Education and Research (Deemed to be University) Salod (H), Wardha, Maharashtra, India,; 2 Department of Kayachikitsa, Mahatma Gandhi Ayurved College Hospital and Research Centre, Datta Meghe Institute of Higher Education and Research (Deemed to be University) Salod (H), Wardha, Maharashtra, India

**Keywords:** Ulnar drift, ulnar deviation, rheumatoid arthritis

## Image in medicine

Rheumatoid arthritis is an autoimmune disease which affects articular and extra-articular structures. It develops slowly in weeks to months, with the onset of signs and symptoms. The patient frequently first experiences stiffness in one or more joints, which is frequently accompanied by discomfort with movement and joint soreness. Although the total number of joints involved varies greatly, the process always ends up being polyarticular, involving five or more joints. A palindromic presentation is another pattern where patients report swelling in one or two joints that may last a few days or weeks before going away completely, only to subsequently return in the same or other joints with an increasing pattern over time. The hands' Proximal Interphalangeal (PIP) and Metacarpophalangeal (MCP) joints, the wrists, and the tiny joints of the feet, especially the Metatarsophalangeal (MTP) joints, are the joints most usually affected. A 62-year-old female patient came to the outpatient department of Mahatma Gandhi Ayurved College Hospital and Research Centre, Salod (H) with all the signs and symptoms of rheumatoid arthritis along with ulnar deviation in both hands. Clinical observation revealed an ulnar deviation.

**Figure 1:**
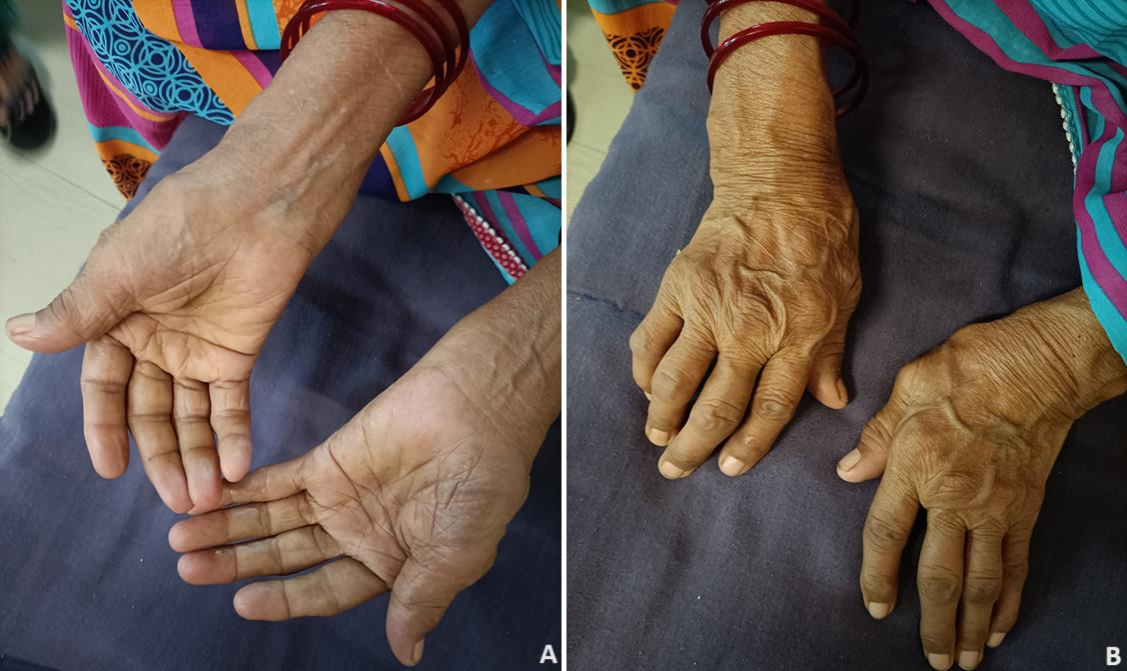
A) ulnar drift - palmer aspect of both hands; B) ulnar drift - dorsal aspect of both hands

